# P03.11. Relaxation response intervention induces respiration and heart rate variability changes in hypertensives

**DOI:** 10.1186/1472-6882-12-S1-P264

**Published:** 2012-06-12

**Authors:** T McConville, K Dusek, J Dusek

**Affiliations:** 1 Penny George Inst. for Health & Healing, Abbott Northwestern Hospital, Allina Health, Minneapolis, USA

## Purpose

Mind/body practices that elicit the relaxation response (RR) have been used for health purposes for thousands of years, but mechanisms of action are not clearly understood. Our research team seeks to identify mechanisms underlying the impact of a mind/body relaxation response intervention on blood pressure. As an extension of that effort, the purpose of the current substudy explores physiologic measurements and heart rate variability (HRV) analysis during elicitation of the relaxation response in hypertensive adults.

## Methods

The current dataset includes 34 subjects (aged 50-75) half of whom were randomized to either an eight week relaxation response (RR) intervention or eight week health education (HE). HRV, heart and respiration rate were collected by ECG and respiratory band in a controlled laboratory setting before and after subjects completed their eight week intervention. Both 75 minute pre and post sessions were exclusively conducted in the morning and included the following periods: habituation, baseline, CD listening and rest. During the pre session CD listening period, all subjects listened to a health education recording. Whereas in the post session CD listening period, RR subjects elicited the RR through audio instructions and HE subjects listened to another health education recording.

## Results

Preliminary results indicate a 19% decrease in respiration rate during acute RR elicitation, without influence on heart rate. However, there was a significant increase in HRV as measured by SDNN, and RMSSD (five minute increments). Both respiration rate and HRV response persisted for approximately 10 minutes beyond RR elicitation. In contrast, HE subjects did not exhibit significant changes to respiration rate, heart rate or HRV measures.

## Conclusion

Hypertensive patients receiving RR training exhibited significant reductions in respiration rate and increase in HRV, whereas HE subjects did not. These types of physiological findings may assist in our eventual exploration of blood pressure changes from the parent study.

